# YMO1 suppresses invasion and metastasis by inhibiting RhoC signaling and predicts favorable prognosis in hepatocellular carcinoma

**DOI:** 10.18632/oncotarget.10866

**Published:** 2016-07-27

**Authors:** Rui-Min Chang, Lei Pei, Feng Fang, Jiang-Feng Xu, Hao Yang, Chao-Hui Zuo, Jian-Hua Zhou, Geng-Qiu Luo, Lian-Yue Yang

**Affiliations:** ^1^ Liver Cancer Laboratory, Xiangya Hospital, Central South University, Changsha 410008, China; ^2^ Department of Pathology, Xiangya Hospital, Central South University, Changsha 410008, China; ^3^ Department of Surgery, Xiangya Hospital, Central South University, Changsha 410008, China; ^4^ Department of Abdominal Surgical Oncology, Affiliated Cancer Hospital of Xiangya School of Medicine, Central South University, Changsha, Hunan, China

**Keywords:** YMO1, RhoC, PAX5, hepatocellular carcinoma, metastasis

## Abstract

Previous studies have shown that 4.1 proteins, which are deregulated in many cancers, contribute to cell adhesion and motility. Yurt/Mosaic eyes-like 1 (YMO1) is a member of 4.1 protein family but it is unclear whether YMO1 plays a role in tumor invasion. This study aimed to investigate the effects of YMO1 on hepatocellular carcinoma (HCC) and attempted to elucidate the underlying molecular mechanisms. YMO1 expression in HCC tissues and its correlation with clinicopathological features and postoperative prognosis was analyzed. The results showed that YMO1 was down-regulated in the highly metastatic HCC cell line and in human tumor tissues. Underexpression of YMO1 indicated poor prognosis of HCC patients. Restoration of YMO1 expression caused a significant decrease in cell migration and invasiveness *in vitro*. *In vivo* study showed that YMO1 reduced liver tumor invasion and metastasis in xenograft mice. YMO1 directly inhibited RhoC activation. YMO1 expression in HCC was regulated by PAX5. Analysis of YMO1 expression levels in human HCC patients revealed a significant correlation of YMO1 expression with PAX5 and RhoC. Our findings revealed that YMO1 predicts favorable prognosis and the data suggest that YMO1 suppresses tumor invasion and metastasis by inhibiting RhoC activity.

## INTRODUCTION

Hepatocellular carcinoma (HCC) is one of the most common cancers and ranks the second leading cause of cancer death in men and the sixth in women over the world [1]. Over decades of extensive studies demonstrate that the capability of cell migration plays an essential role in the recurrence and metastasis of a wide variety of tumors including HCC [2]. As a result, many molecules relevant to cell migration have been found to be implicated in tumor invasion and correlated with poor prognosis for human HCCs [3–5]. While these discoveries are a great step forward to understand the mechanisms underlying poor prognosis of HCC [2].

The family of 4.1 proteins consists of the eponymous 4.1R protein (EPB41) initially identified in erythrocytes, 4.1N (EPB41L1), 4.1G (EPB41L2), 4.1B (EPB41L3) and the less closely related members NBL4 (EPB41L4A), EHM2 (EPB41L4B) and YMO1 (EPB41L5) [6]. They form nodes in the cell cortex by connecting other components of the cortical cytoskeleton such as spectrins, actin and transmembrane adhesion proteins, receptors and transporters. Therefore, 4.1 proteins contribute to the organization of cell polarity, adhesion and motility, and regulate the transport and response for growth factors [7, 8]. As such, altered expressions for 4.1 proteins are commonly noted in many types of cancers such as epithelial ovarian cancer or gliomas [9, 10]. There is also evidence suggesting a role for the 4.1 related proteins ezrin in HCC tumorigenesis [11], but the role of other 4.1 proteins such as YMO1 in HCC tumorigenesis remains largely unknown. We herein hypothesize that YMO1 also has some roles in HCC progression.

To test the hypothesis above, we firstly examined the YMO1 expression in 223 HCC patients with long-term follow-up studies. We found that low YMO1 expression predicts poor prognosis of HCC patients. It's expression is correlative with metastatic clinical pathological characteristics of patients. So we further explored the role of YMO1 on metastasis of HCC *in vivo* and *in vitro*. All together, our data support that YMO1 functions as a novel tumor suppressor to prevent HCC invasion and metastasis.

## RESULTS

### YMO1 is significantly reduced in HCC tissues

To obtain the evidence supporting a role for YMO1 (EPB41L5) in the recurrence and metastasis of HCC, we first examined its expression in HCC tissue and the adjacent nontumoral liver tissue (ANLT) in the same patient by real-time PCR, in which 30 HCC patients were randomly selected for the study. Remarkably, a significant reduction for YMO1 expression was consistently detected in all HCC tissues as compared with that of the ANLT tissues in all patients examined (*P*<0.001, Supplementary Figure S1A). This result prompted us to examine the expression profile of other members for the 4.1 protein family using the same approach. A reduced expression for EPB41L3 (*P*<0.001, Supplementary Figure S1B) and an increased expression for EPB41L4B (*P*=0.002, Supplementary Figure S1C) were also noticed in HCC tissues. However, we did not detect a discernable change for the EPB41, EPB41L1, EPB41L2 and EPB41L4A mRNA between HCC and the ANLT samples (Supplementary Figure S1D to S1G).

**Figure 1:**
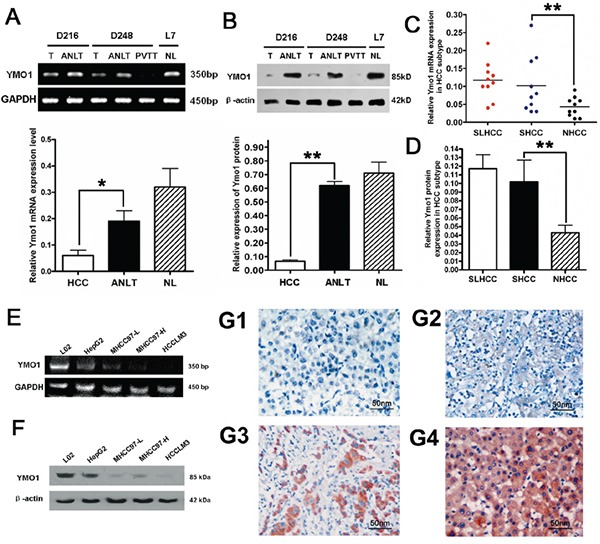
Expression level of YMO1 in HCC tissues and HCC cell lines **A.** The mRNA level of YMO1 was determined by RT-PCR in HCC tissues (n=30) and in ANLTs (n=30). **B.** The YMO1 protein level was determined by western blot. The representative western blots of normal liver tissues, HCC tissues, ANLT and portal vein tumor thrombosis (PVTT) are shown. The average value of β-actin protein was used to normalize to the level of YMO1 protein. **C.** The YMO1 mRNA levels in SLHCC (n=10), SHCC(n=10) and NHCC (n=10) were compared. **D.** Three subtypes of HCC tissues were compared for the YMO1 protein expression. **E.** The relative expression level of YMO1 mRNA in normal cell line and HCC cell lines, determined by RT-PCR. **F.** The expression levels of YMO1 protein correlated with the invasion potential of HCC cell lines. **G1-G3.** Representative image for YMO1 immunohistochemistry staining in HCC tissues. **G4.** Representative image for YMO1 diffuse strong staining in ANLT. Original magnification×400. *, *P* < 0.05; **, *P* < 0.01.

YMO1 expression levels in 30 pairs HCC and ANLT tissues of were analyzed and compared in parallel with that of liver tissues isolated from normal controls. Consistent with the above results, mRNA and protein of YMO1 were significantly downregulated in HCC tissues. (*P*<0.05; Figure 1A, 1B). Interestingly, when stratified according to HCC subtypes, we found although the YMO1 expression in SHCC and SLHCC was not statistically different, expression in NHCC was lower than that in SHCC and SLHCC (Figure 1C, 1D).

We further examined the differences of YMO1 expression between four HCC cell lines and a normal liver cell line (L02 cells) by RT-PCR (Figure 1E) and western blot (Figure 1F) analysis. Of note, the extent for reduction of YMO1 expression correlated with the metastatic potential (Figure 1E, 1F), since HCCLM3 cells possess the highest capability for metastasis, while HepG2 cells have the least capacity for metastasis [5, 12].

Immunohistostaining of YMO1 positive expression showed cytoplasmic location and brown staining in cells (Figure 1G1-1G4). The YMO1 expression was observed in 197 of 223 cases (88.3%) in ANLT sample and 132 of 223 cases (59.2%) in HCC sample, showing cytoplasmic patterns. Immunohistochemistry of YMO1 showed that none or few cells showing positively-stained cytoplasm was detected in HCC tissues (Figure 1G1-1G3). We observed diffuse strong brown in ANLT(Figure 1G4) or cirrhotic liver tissues around HCC tumor lesion (Supplementary Figure S2). 178 of 223 (79.8%) tumor tissues were weakly stained relatively to the ANLT. However, immunohistochemistry of YMO1 didn't show significantly different positively-staining in HCC lesion of different differentiation grades.

### Correlations of YMO1 expression with clinicopathologic characteristics and prognosis of HCC

Subsequently, the association of YMO1 expression with the clinicopathologic features of HCC was analyzed. A total of 223 HCC cases were collected in two independent hospitals as described (Supplementary Figure S3). The HCC patients were stratified into low expression group and high expression group according to result of immunohistochemistry. The clinicopathological characteristics of patients in training cohort and validation cohort were supplied in Supplementary Table S1. The correlations of YMO1 expression with clinicopathologic characteristics and prognosis of HCC were analyzed. It's found that, in training cohort, the YMO1 expression was related with tumor nodule number, vascular invasion and TNM (Table 1). And in validation cohort, the YMO1 expression was related with tumor nodule number, capsular formation, vascular invasion and TNM (Supplementary Table S2). Due to these characteristics are recurrence related indexes, so we speculated that YMO1 maybe also a prognostic marker for HCC after liver resection.

**Table 1:** The correlations of YMO1 with clinicopathological features of HCC in training cohort

Clinicopathologic variable	YMO1			
	n	Low expression	High expression	*P*
Gender				
Female	26	13	13	
Male	127	77	50	0.316
Age(year)				
≤60	121	71	50	
>60	32	19	13	0.943
AFP				
<20 ng/ml	50	30	20	
≥20 ng/ml	103	60	43	0.837
HBsAg				
Negative	35	21	14	
Positive	118	69	49	0.872
Liver cirrhosis				
Absence	46	24	22	
Presence	107	66	41	0.273
Tumor size(cm)				
≤5	64	32	32	
>5	89	58	31	0.060
Tumor nodule number				
Solitary	84	41	43	
Multiple(≥2)	69	49	20	**0.005**
Capsular formation				
Presence	89	47	42	
Absence	64	43	21	0.075
Edmondson-Steiner grade				
I-II	85	46	39	
III-IV	68	44	24	0.186
Vascular invasion				
Absence	95	50	45	
Presence	58	40	18	**0.046**
TNM				
I	74	36	38	
II-III	79	54	25	**0.013**
BCLC staging				
0-A	81	48	33	
B-C	72	42	30	0.908
Child-Pugh staging				
A	103	58	45	
B	50	32	18	0.365

**Figure 2:**
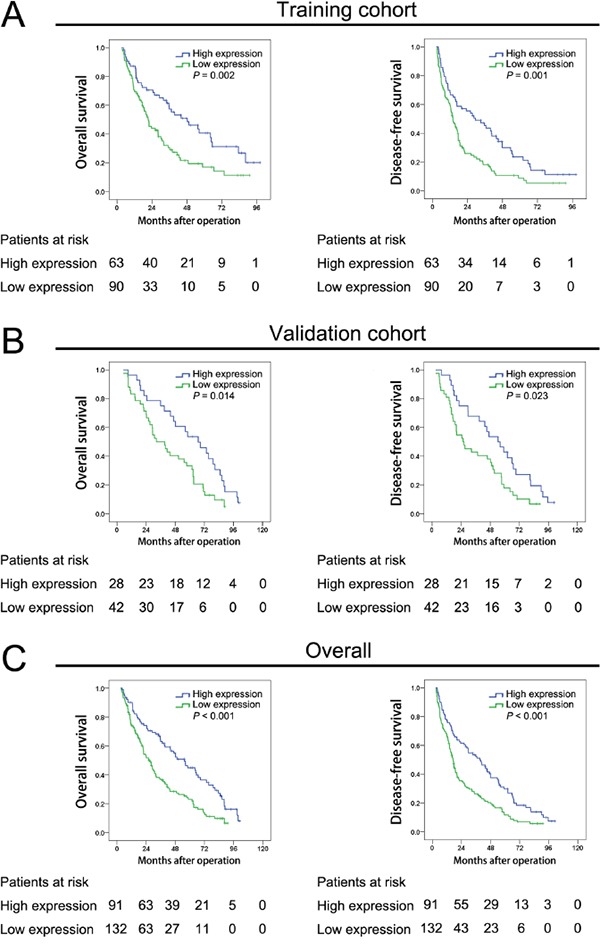
Low YMO1 expression indicated relative poor postoperative prognosis of HCC patients All HCC cases included in this study were stratified into training cohort and validation cohort according to the progress supplied in Supplementary Figure S1. The Kaplan-Meier curves showed the overall survival and disease-free survival of patients with high or low YMO1 expression in **A.** training cohort (n=70) and **B.** validation cohort (n=153). And these data were further validated in **C.** overall cases including training cohort and validation cohort (n=223).

Kaplan-Meier curve revealed the overall survival and disease-free survival of high YMO1 expression group and low YMO1 expression group. The overall survival of the patients with high YMO1 expression group in training cohort were better than patients with low YMO1 expression (*P* = 0.002). The disease-free survival of the patients with high YMO1 expression group in training cohort were also better than patients with low YMO1 expression (*P* = 0.001) (Figure 2A). Similarly, in validation cohort, the overall survival and disease-free survival for HCC patients with high YMO1 expression are much better than patients with low YMO1 expression (*P* = 0.014 and *P* = 0.023, respectively) (Figure 2B). To further demonstrate this result, the data of patients in two cohorts were unified and analyzed. The overall survival and disease-free survival (Figure 2C) of high YMO1 group were better than those of low YMO1 group (*P*<0.001), which are consistent with previous finding (Figure 2A, 2B).

Subsequently, Cox regression was also performed to analyze whether YMO1 is an independent risk factor of HCC prognosis. The result showed that tumor nodule number, vascular invasion and YMO1 expression are independent risk factor for overall survival (Table 2). And liver cirrhosis, tumor nodule number, capsular formation, vascular invasion and YMO1 expression are also independent risk factor for disease-free survival of HCC (Supplementary Table S3). Similarly, univariate and multivariate analyses in validation cohort showed that tumor nodule number, vascular invasion, TNM and YMO1 expression are independent risk factors for overall survival of HCC (Supplementary Table S4). And tumor nodule number, vascular invasion, TNM and YMO1 expression are independent risk factors for disease-free survival of HCC (Supplementary Table S5). These results together revealed that low YMO1 expression indicated relative worse prognosis of HCC than high YMO1 expression, implicating YMO1 perhaps participates in HCC progression.

**Table 2:** Univariate and multivariate analysis of factors associated with overall survival in training cohort

Variable		Univariate analysis		Multivariate analysis	
	n	RR(95%CI)	*P*	RR(95%CI)	*P*
Gender					
Female	26	1			
Male	127	1.138(0.684-1.895)	0.619	n.a.	n.a.
Age(year)					
≤60	121	1			
>60	32	1.248(0.788-1.975)	0.345	n.a.	n.a.
AFP					
<20 ng/ml	50	1			
≥20 ng/ml	103	1.230(0.849-1.779)	0.274	n.a.	n.a.
HBsAg					
Negative	35	1			
Positive	118	1.252(0.823-1.901)	0.294	n.a.	n.a.
Liver cirrhosis					
Absence	46	1			
Presence	107	1.436 (0.93.-2.210)	0.100	n.a.	n.a.
Tumor size(cm)					
≤5	64	1			
>5	89	1.190(0.801-1.767)	0.390	n.a.	n.a.
Tumor nodule number					
Solitary	84	1		1	
Multiple(≥2)	69	1.572 (1.062-2.331)	**0.024**	1.675 (2.506-41.122)	**0.012**
Capsular formation					
Presence	89	1		1	
Absence	64	1.663(1.124-2.459)	**0.011**	1. 344(0.891-2.026)	0.159
Edmondson-Steiner grade					
I-II	85	1			
III-IV	68	1.176(0.795-1.739)	0.419	n.a.	n.a.
Vascular invasion					
Absence	95	1		1	
Presence	58	2.290(1.535-3.419)	**<0.001**	2.059(1.362-3.112)	**0.001**
TNM					
I	74	1			
II-III	79	1.346(1.031-1.756)	**0.029**	1.280(0.979-1.674)	0.103
BCLC staging					
0-A	81	1		1	
B-C	72	1.636(1.122-2.387)	**0.011**	1.334(0.953-1.867)	0.077
Child-Pugh					
A	103	1			
B	50	1.329(0.885-1.994)	0.170	n.a.	n.a.
YMO1 expression					
high	63	1		1	
Low	90	1.911(1.267-2.882)	**0.002**	1.862(1.221-2.841)	**0.004**

### Ectopic YMO1 expression inhibits HCC cell invasion and migration

**Figure 3:**
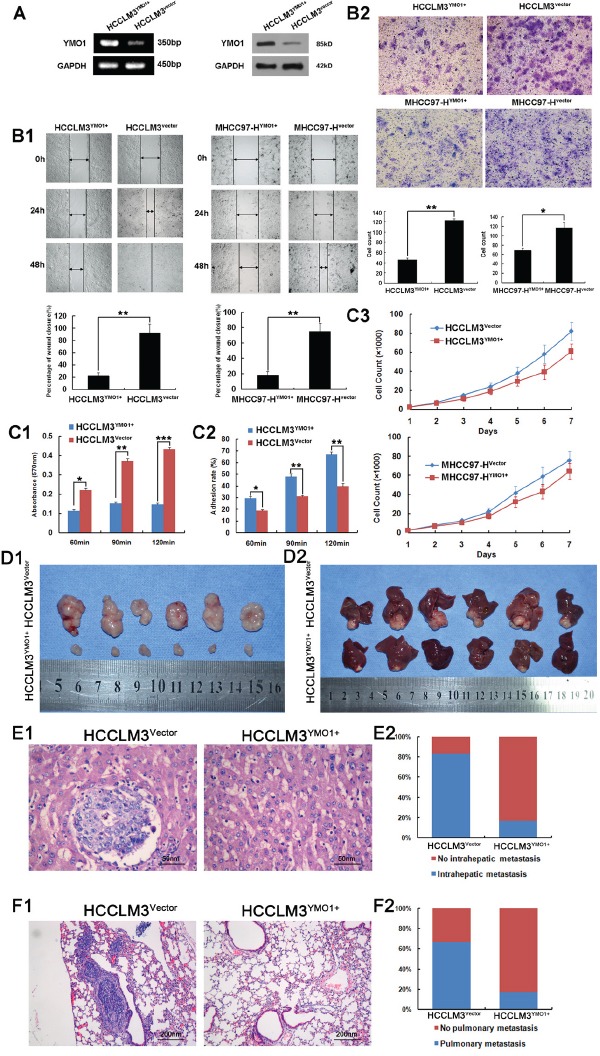
Overexpression of YMO1 suppresses HCC cell migration and invasive potential *in vitro* and *in vivo* **A.** The expression efficiency of pcDNA3- YMO1 was evaluated by RT-PCR and Western blot analysis. **B1.** The indicated cell lines were scratched and wound closures were measured for 0, 24 and 48 hours culture. **B2.** The indicated cells that invaded through matrigel-coated transwell were stained with crystal violet. **C1.** Cells were inoculated in fibronectin-coated plastic dishes and absorbance at 570 nm was measured. **C2.** Monolayer 10^6^ cells were plated on monolayer of HCC cell. After incubation and elution, rate of adherence was measured. **C3.** HCC cells were subjected to proliferation rate analysis. The cell numbers are the medians of 3 independent experiments (mean±SD). **D1.** HCC subcutaneous xenograft model with empty vector and with pcDNA3- YMO1 transfected HCCLM3 cells. **D2.** Assessment of tumor growth promotion in orthotopic xenograft of HCCLM3^YMO1+^ and HCCLM3^vector^ group. The representative H&E staining images of **E1.** intrahepatic metastasis and **F1.** pulmonary metastasis. The percentage of mice with or without intrahepatic and pulmonary metastasis was calculated and compared.*, *P* < 0.05; **, *P* < 0.01; ***, *P* < 0.001.

To functionally characterize YMO1 in HCC cell invasion and migration, we performed wound healing and transwell assays. The HCC cells with highly invasive potential (HCCLM3 and MHCC97-H) and HCC cells with lowly invasive potential (HepG2) were transfected with corresponding plasmid, and the YMO1 expression was confirmed by RT-PCR and western blot analysis (Figure 3A, Supplementary Figure S5A). The cells were next subjected to wound-healing migration assay. Remarkably, a significant delay for would healing was noticed in cells transfected with pcDNA-YMO1 when compared with cells transfected with control vector (Figure 3B1). In addition, enforced YMO1 expression has also decreased the capacity of invasiveness for HCCLM3 and MHCC97-H cells as assessed by the transwell assays (Figure 3B2). Furthermore, YMO1 expression suppressed cell-ECM adhesion (Figure 3C1), which was associated with enhanced cell-cell adhesion (Figure 3C2). Besides, YMO1 may also slightly inhibited HCCLM3 or MHCC97-H proliferation (Figure 3C2). However, YMO1 didn't significantly induce apoptosis of HCC cells (Supplementary Figure S4). And we performed these assays again in HepG2 cells and HepG2^shYMO1^ cells. We also found that inhibition of YMO1 promoted invasion and migration of HCC cells (Supplementary Figure S5B-S5F).

### YMO1 suppresses implanted tumor growth and metastasis

Consistent with the *in vitro* data, the subcutaneous tumor size was significantly smaller in mice implanted with YMO1-transfected cells when compared with that of control vector transfected cells (*P*<0.01, Figure 3D1). Similarly, the growth of tumors in mouse liver orthotopic cancer xenograft model originally formed from YMO1 transfected cells were significantly slower than that of control vector transfected cells (Figure 3D2). We also evaluated the differences of intrahepatic metastasis and pulmonary metastasis (Figure 3F). Consistently, the rates for intrahepatic metastasis (*P*<0.001, Figure 3E1-3E2) and pulmonary metastasis (*P*<0.001, Figure 3F1-3F2) were significantly lower for mice in YMO1-transfected group as compared with that of control vector group. Taken together, our data support that YMO1 is potent to suppress tumor formation and metastasis.

### YMO1 interacts with RhoC and suppresses its activity

**Figure 4:**
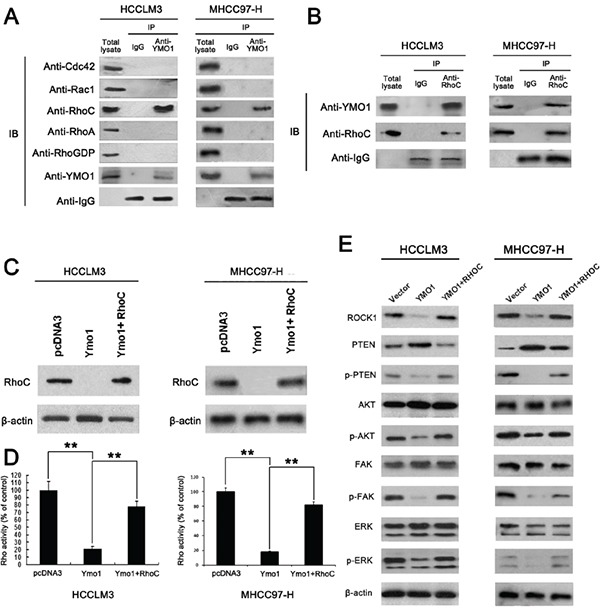
YMO1 suppresses cell motility and invasion by interacts with RhoC and suppressing Rho-GTPase activity **A.** YMO1 binding to Rho GTPase components in HCCLM3 and MHCC97-H cells. Cell lysates were immuno-precipitated with rabbit IgG or rabbit anti-YMO1 antibody, and the presence of each component in the precipitates was blotted with the antibody against each molecule. **B.** Results of co-immunoprecipitation. The cell lysate was immuno-precipitated by anti-RhoC antibody and immuno-blotted against anti-YMO1 antibody. **C.** RhoC expression in HCCLM3 and MHCC97-H only transfected YMO1 vector or cotransfected YMO1 and RhoC vector and **D.** Levels of total and GTP-bound RhoC were determined in two HCC cell lines. Rho activity in cells transfected with pcDNA3 empty vector, pcDNA3 containing YMO1 or pcDNA3 containing YMO1 and RhoC. Rho activity was normalized to the relative band intensities on the immunoblot measured by densitometry. **E.** ROCK1, PTEN, p-PTEN, AKT, p-AKT, FAK, ERK and p-ERK protein expression in these HCC cells. **, *P* < 0.01.

To investigate the mechanisms by which YMO1 suppresses tumor formation and metastasis, we examined potential signaling pathways YMO1 might be involved. Given that the Rho GTPases are important effectors to control actin-dependent cell motility or invasion [13], we tested whether YMO1 inhibited Rho family GTPase expression. Co-immunoprecipitation assays against Rac1, Cdc42, RhoA, RhoC or RhoGDI, respectively, were performed. Indeed, we detected an interaction between YMO1 and RhoC as evidenced by the presence of an YMO1 reactive band from the RhoC precipitates (Figure 4A, 4B).

The above results prompted us to examine the effect of YMO1 interaction on RhoC expression and enzymatic activity. We first examined the impact of YMO1 on RhoC expression. We transfected HCCLM3 cells with a RhoC plasmid along with either an YMO1 construct or a control vector, and then analyzed RhoC protein levels by western blot analysis. Interestingly, YMO1 expression significantly suppressed RhoC expression (Figure 4C). In line with this result, RhoC GTPase activity in YMO1 co-transfected cells was much lower than that of control vector co-transfected cells (Figure 4D). Furthermore, YMO1 expression suppressed RhoC mediated invasion (Supplementary Figure S6A), migration (Supplementary Figure S6B) and cytoskeletal reorganization (Supplementary Figure S6C), both in HCCLM3 cells and MHCC97-H cells.

The next key question is whether YMO1-mediated suppression of tumor invasion and metastasis depends on its inhibitory effect on RhoC activity. To address this question, we used a siRNA specific for RhoC for the study. Significantly, knockdown of RhoC by siRNA almost completely abolished the ability of YMO1 to promote cell invasion (Supplementary Figure S6D) and migration (Supplementary Figure S6E). Collectively, these results strongly suggest that YMO1 inhibits RhoC expression and its enzymatic activity, through which it suppresses tumor formation and metastasis.

### The RhoC/ROCK1 pathway plays an essential role in YMO1 function

Downstream signaling pathways of RhoC were analyzed by expression of ROCK1 (Rho-associated coiled-coil-containing protein kinase) and phosphorylated forms of phosphatase and tensin homolog deleted on chromosome ten (PTEN), V-akt Murine Thymoma Viral Oncogene Homolog (AKT), focal adhesion kinase-1 (FAK-1), and extracellular signal-regulated kinase (ERK) by western blot. The ROCK1 was observed a significantly decrease in HCCLM3^YMO1+^, whereas there was no discrepancy between HCCLM3^vector^ and co-transfect group HCCLM3^YMO1+RhoC^. Moreover, YMO1 overexpression repressed RhoC/ROCK1 downstream pathways p-AKT, p-FAK and p-ERK signal in HCC cells transfected YMO1 vector. Interestingly, phosphorylation of PTEN was observed to be suppressed after overexpression of YMO1 in HCCLM3 (Figure 4E). Taken together, these data show requirement RhoC/ROCK1 pathway in YMO1-mediated suppression of invasion and adhesion.

### PAX5 increases YMO1 transcription

**Figure 5:**
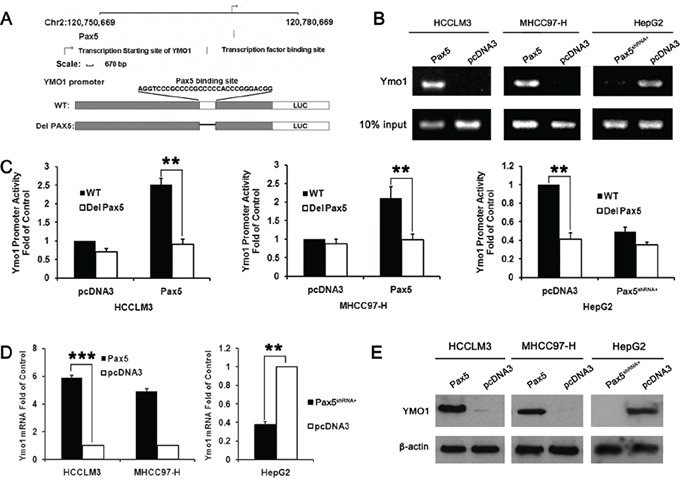
Exogenous expression of PAX5 induced up-regulation of YMO1 expression through transcriptional activation **A.** Decipherment of DNA Elements Database (DECODE) predicts PAX5 as a relevant transcription factors and transcription factor binding sites in YMO1 gene promoter. Diagrammatic sketch show the construction for wide type (WT) and PAX5 binding site deletion (Del PAX5) luciferase vectors. **B.** HCCLM3, MHCC97-H and HepG2 cells were cross-linked with formaldehyde and lysed. The soluble chromatin was immunoprecipitated with the anti-PAX5 antibody. Primers were designed to detect the promoter region of YMO1. The immunoprecipitated YMO1 DNA was detected after transfected with PAX5-pcDNA3 by semiquantitative ChIP-PCR assay. **C.** Dual luciferase reporter assay of pGL3-YMO1 in HCC cells transfected PAX5 or PAX5^shRNA+^ vector or the empty vectors. **D.** mRNA and **E.** protein levels of YMO1 in HCC cells transfected with the PAX5 or PAX5^shRNA+^ vector or the empty vector. **, *P* < 0.01; ***, *P* < 0.001.

We next sought to investigate how YMO1 expression is regulated. For this purpose, we performed bioinformatics analysis of the *YMO1* promoter, and found a highly conserved PAX5 binding site at the *YMO1* promoter (Figure 5A). PAX5 is also one of factors inversely correlated with tumor nodule number, capsule formation, vascular invasion and TNM Stage (Supplementary Table S6). To determine whether PAX5 binds to the *YMO1* promoter, we carried out ChIP assays using a PAX5 antibody. As shown in Figure 5B, PCR amplification of the ChIP products using a pair of primers flanking the putative PAX5 binding site yielded a corresponding positive band in HCCLM3 cells transfected PAX5 vector, while no products were detected in the precipitates from HCCLM3 cells transfected control vector. To determine that PAX5 increases YMO1 transcription after binding to its promoter, we did promoter reporter assays. HCCLM3 cells were transfected with a pGL3-YMO1 reporter along with a wide type(WT) *YMO1* promoter. An YMO1 reporter in which the PAX5 binding site was mutated (Del PAX5) was used as a control (Figure 5A). As expected, the reporter activity was significantly higher in the cells transfected with the wild-type YMO1 reporter but not the mutant (Figure 5C), demonstrating that PAX5 increases YMO1 transcription. These results were further confirmed by real-time PCR and western blot analysis, in which the cells transfected with PAX5 showed significantly higher levels of YMO1 mRNA (Figure 5D) and protein (Figure 5E) as compared with that of cells transfected with an empty vector. Besides, our data also showed that there isn't any mutation in the PAX5-binding domain within the promoter of YMO1 (Supplementary Figure S7). All together, our data suggest that PAX5 binds to the *YMO1* promoter, and increases YMO1 expression. Moreover, to further validate these results, we also did the “loss of function study”. HepG2 cell line was transfected with PAX5 shRNA and YMO1 expression level was examined. ChIP assays showed that PAX5 binds to the YMO1 promoter in pcDNA3 group. But after PAX5 was knockout, YMO1 promoter couldn't be found in immunoprecipitate (Figure 5B). The reporter activity in HepG2 cells transfected with PAX5 shRNA was significantly lower than that in cells transfected with empty control vector. (Figure 5C) The expression levels of YMO1 mRNA and protein were also significantly lower in HepG2 cells transfected with PAX5 shRNA than that in cells transfected with pcDNA3 vector (Figure 5D, 5E).

### Correlation analysis of YMO1, PAX5 and RhoC expression in HCC samples

**Figure 6:**
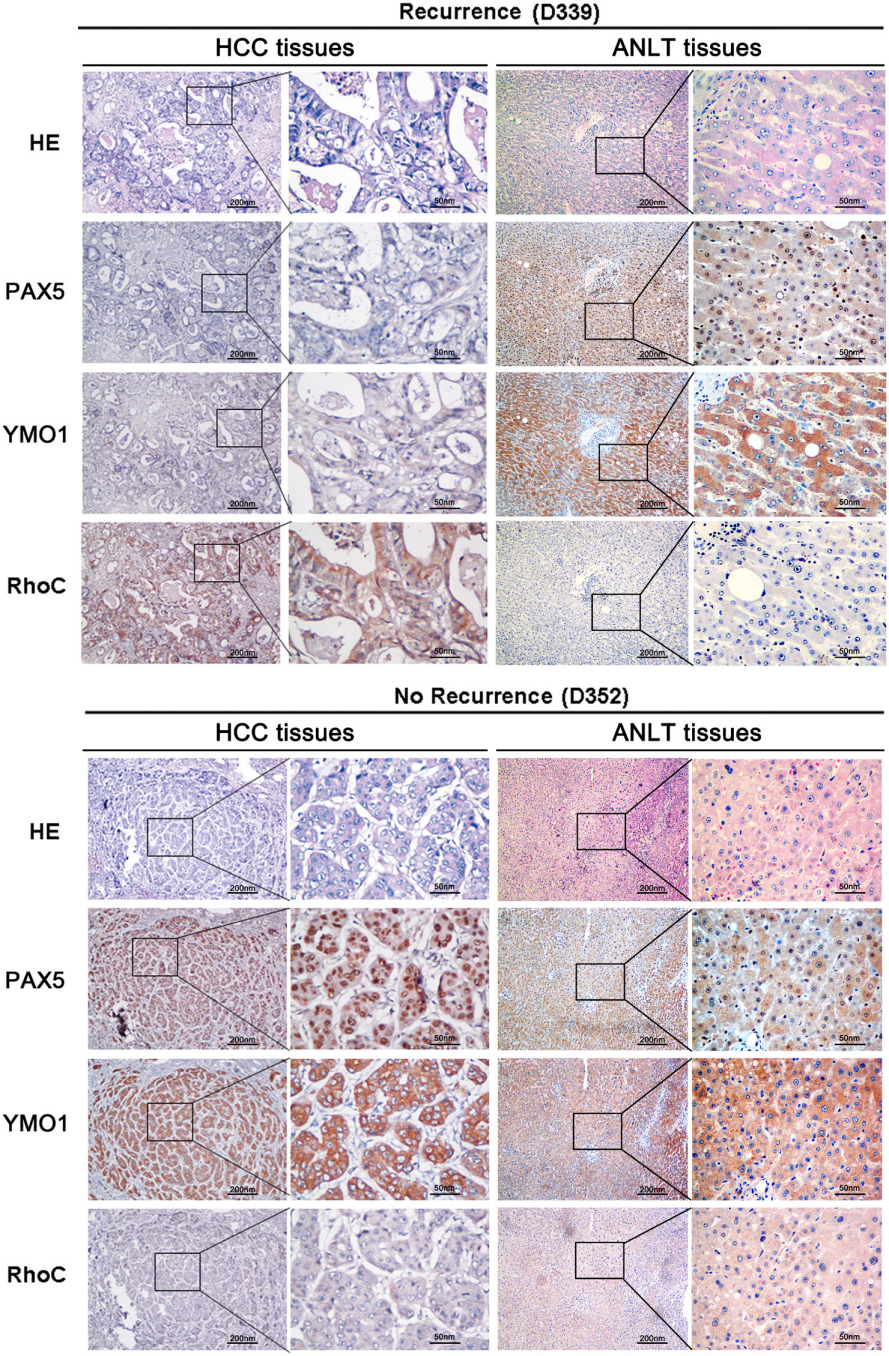
Protein levels of YMO1, PAX5 and RhoC in human HCC samples Representative immunohistochemical staining of PAX5, YMO1 and RhoC in serial sections of samples from a patient with recurrence (sample No. D339) and a patient without recurrence (sample No. D352).

To determine the clinical relevance of association of YMO1, RhoC and PAX5 in cancer invasion and metastasis, we validated the experimental results in tumor tissues derived from 153 HCC patients in training cohort. We performed correlation analysis between the expression levels for YMO1, PAX5 and RhoC and the presence of recurrence of HCC based on immunohistochemistry staining as described (Figure 6). It is noteworthy that the expression levels for YMO1 positively correlated with PAX5 expressions in all HCC samples analyzed (*P* = 0.004, Supplementary Table S7), in which the absence of recurrence was associated with the high levels of YMO1 and PAX5 expressions in HCC samples. On the contrary, YMO1 expression inversely correlated with RhoC expression (*P* = 0.009), in which HCC with recurrence were generally associated with increased RhoC expression.

## DISCUSSION

The 4.1 proteins family is characterized by FERM (Four-point-one, Ezrin, Radixin, Moesin) domains [14], many of which have related functions in influencing the biologic characteristics of tumor cells, as reported mainly with regard to cancer progression [9, 15]. However, the role of 4.1 proteins family in human HCC is currently unknown. YMO1, which belongs to the 4.1 proteins family, has been reported to contribute to the regulation of cell adhesion and motility during embryos development [16, 17]. However, little is known of the role for YMO1 in the development of human HCC. In this study, we firstly investigated the expression of YMO1 in HCC tissues and found that YMO1 expression decreased significantly in most HCC tissues tested, suggesting a potential involvement of YMO1 in the development of HCC. It has been reported that YMO1 is a key regulator of epithelial cell architecture and interacts directly with cytoplasmic complex components via PDZ domains [18]. Hence, it suggests that YMO1 is distinct because of its unique C-terminal PDZ domains.

It is reported that some proteins with the FERM domain are able to interact with Rho-family GTPases [19]. YMO1 is a type of mammalian Yurt orthologs which containing a FERM domain [16, 20]. Having observed YMO1-dependent suppression of cell motility or invasion, we explored a potential interaction of YMO1 with Rho family GTPases [4]. Our result indicates that YMO1 is directly binding to RhoC. Furthermore, overexpression of YMO1 resulted in inhibition of cell migration and invasion by inhibiting RhoC activity and expression. However, YMO1 didn't increased ubiquitination of RhoC (Supplementary Figure S8). Our previous study has indicated that RhoC expression was remarkably increased in HCC tissues and was associated with metastasis of HCC [21]. Rho-GTPases, members of Ras superfamily of small GTPases, shuttle between inactive GDP-bound and active GTP-bound form and exhibit intrinsic GTPase activities [22]. Activation of the RhoC GTPase leads to the assembly of the actin-myosin contractile filaments into focal adhesion complexes that promote cell polarity and facilitate motility [23]. Suppression of RhoC expression resulted in inhibition of invasion and migration [21]. These results are consistent with our data showing the role of YMO1.

Rho proteins and ROCK proteins are important regulators of cell migration, proliferation and apoptosis [24]. Activated ROCK1 by Rho GTPase family member binds and phosphorylates AKT, PTEN, FAK [25, 26]. Previous studies have demonstrated that phosphorylation of AKT plays a critical role in intrahepatic metastasis in an orthotopic implantation model of HCC and this regulation is dependent on Rho/ROCK1 activation [27]. Our current studies suggest that p-AKT is responsible for YMO1-mediated Rho/Rock1 activation and cell invasion/metastasis. And we also found the remarkable function of YMO1 in phosphorylation of PTEN. Ample evidences suggest that phosphorylation improves PTEN stability but attenuates PTEN function, which will leads to the activation of AKT [28]. Furthermore, we also observed a role of FAK in YMO1-regulated Rho/Rock1 activation, resulting in inhibition of ERK activity in HCC cells, consistent with previous reports that Rho/Rock1 activates ERK signal, promoting angiogenesis and invasion of HCC [29]. Taken together, this study clearly demonstrates a crucial role for YMO1 in the induction of cell invasion and migration through negatively regulating the RhoC/ROCK1 pathway and downstream AKT and ERK signal.

With the aid from decipherment of DNA Elements Database, we predicted and verified that YMO1 transcription was increased by the transcription factor PAX5. In line with our data, a research suggests that PAX5 as a novel tumor suppressor in human HCC and ectopic expression of PAX5 mediates upregulation of the tumor suppressor pathway, including the pathway of p53 [30]. Our data suggest that PAX5 functions as a transcription factor of YMO1, further supporting a tumor suppressor role of PAX5.

To our knowledge, our present study is the first report that decreased expression of YMO1 predicted poor prognosis and inhibited invasion and metastasis of HCC by suppressing RhoC signaling. YMO1 may be of great value in predicting the prognosis of HCC patients and serve as a potential therapeutic target for HCC.

## MATERIALS AND METHODS

### Wound healing, transwell and MTT assay

Wound healing, transwell and MTT assays were conducted as described previously [31]. All assays were carried out in triplicates.

### Patients and specimens

From January 2003 to December 2007, 378 patients with live tumor underwent surgical resection in Department of Surgery, Xiangya Hospital of Central South University (CSU) and 283 patients with live tumor underwent surgical resection in Department of Abdominal Surgical Oncology, The Affiliated Cancer Hospital of Xiangya School of Medicine, CSU were collected. Among them, 160 patients were randomly selected into training cohort from Xiangya Hospital and 80 patients were randomly selected into validation cohort from The Affiliated Cancer Hospital. 7 patients (6 patients with cholangiocarcinoma, 1 patients with haeangioma) in training cohort and 10 patients (7 patients with cholangiocarcinoma, 3 patients with haeangioma) in validation cohort were excluded from this study. At last, 153 HCC patients were in training cohort and 70 HCC patients were in validation cohort (Supplementary Figure S3). Matched HCC tissue and the adjacent non-tumor liver tissue (ANLT) specimens were obtained from these HCC patients Normal liver tissues were obtained from 6 patients with giant hemangioma during hepatic resection. Tissue specimens were fixed by formalin immediately upon collection and then paraffin-embedded. The pathological diagnosis for all the cases was made by at least two Board Certified pathologists working at the Department of Pathology in the Xiangya Hospital of CSU. Among which, 30 subjects with matched fresh HCC tissues and ANLTs were randomly selected for real-time PCR and western blot analysis. The 30 HCC patients were divided into three subgroups as follows: SHCC: Small HCC (<5 cm in diameter); SLHCC: This subtype of HCC has just a solitary node, >5 cm in diameter, and grows expansively within an intact capsule or pseudocapsule [32]. NHCC: Nodular HCC, this subtype of HCC has more than 2 nodes. All research protocols strictly complied with REMARK guidelines for reporting prognostic biomarkers in cancer [33]. Prior informed consent was obtained from all patients and the study was approved by the Ethics Committee of Xiangya Hospital of CSU.

### Real-time PCR analysis

Total RNA was extracted from cell lines or frozen tumor specimens using the Trizol reagents (Invitrogen, Carlsbad, CA) according to the manufacturer's instructions. Real-time PCR was performed using a ReverTra Ace-α- Kit (Toyobo, Osaka, Japan) and the SYBR^®^ Green Realtime PCR Master Mix (Toyobo) as described previously. Primers for each target gene are as follows: YMO1-F, 5′-CCT GGA CCT GAT TGA AAG-3′, YMO1-R, 5′-CAG TCT GAA TAG GCA CGA-3′; EPB41-F, 5′-TCT TAA CAT CAA TGG GCA AAT CC-3′, EPB41-R, 5′-CAC AGC ATT GGC ATT ATC TGA GA-3′; EPB41L1-F, 5′-CTT TGA GCG CAC TTC TAG TAA ACG-3′, EPB41L1-R, 5′-GCG CCA GGA AAA TCC TTC AT-3′; EPB41L2-F, 5′-AGC CTT GAT GGA GCA GAG TTC TC-3′, EPB41L2-R, 5′- CCA TCC TCA TCC CGC TTG T-3′; EPB41L3 -F, 5′-CAC CAG ACC CTG CCC AAC T-3′, EPB41L3-R, 5′-TCC GGA CAC GAT GTC ATC TC -3′; EPB41L4A-F, 5′-AGC CCA TAA CAG TGG TGA AGA T-3′, EPB41L4A-R, 5′-TCA TTC TCC TGC CGT ATT CTG T-3′; EPB41L4B-F, 5′-GTT ACG AAG AAC CAG CAC TT-3′, EPB41L4B-R, 5′-TGA CAT TTG GAG AGT GAG GAT G-3′; EPB41L5-F, 5′- GCA GAG TGG CTG CGT GAA AC-3′, EPB41L5-R, 5′-TGT GAA TAA GGA TGC AAT GTC CAG A -3′; and GAPDH-F, 5′-GCA CCG TCA AGG CTG AGA AC-3′, GAPDH-R, 5′-TGG TGA AGA CGC CAG TGG A-3′. Relative expression levels of each gene were normalized by GAPDH. PCR amplifications were carried out by 50 cycles at 95°C for 5 sec, 60°C for 20 sec. The Applied Biosystems 7300 Fast Real-Time PCR System (Applied BioSystems, Foster City, CA) was used for qRT–PCR. The results were analyzed using the 2^−ΔCt^ method with a formula ΔCt=Ct_YMO1_ -Ct_GAPDH_.

### Western blotting

Total proteins were extracted and resolved by 10% SDS-PAGE and then transferred onto PVDF membranes (Millipore, Bedford, MA). The blotted membranes were blocked by milk (5 mg/mL) and then incubated with primary antibodies against YMO1 (1:300), Pax5 (1:1000) and RhoC (1:1000) (Santa Cruz Biotechnology, Santa Cruz, CA), respectively, followed by incubation with a HRP-conjugated IgG (1:3000) (KPL, Gaithersburg, MD). The relative intensities of each band were normalized to the band intensities of β-actin (Sigma-Aldrich, St. Louis, MO).

Antibodies used in western analysis: mouse mAb for AKT (cat. 2920S) (Cell Signaling Technology, Beverly, MA) and rabbit mAb for phospho-ser473-AKT (cat. 4060) (Cell Signaling Technology); mouse mAb for phosphor-ERK1/2 (cat. Sc-7383) (Santa Cruz Biotechnology, Santa Cruz, CA); rabbit polyclonal antibody for ERK (cat. Sc-94) and FAK (cat. Sc-557) and phosphor-PTEN (cat. Sc-31714) (Santa Cruz Biotechnology, Santa Cruz, CA); goat polyclonal antibody for phosphor-FAK (cat. Sc-11766) and PTEN (cat. Sc-6818) (Santa Cruz Biotechnology, Santa Cruz, CA).

### Immunohistochemistry (IHC)

All HCC tissues were first evaluated by two certified histopathologists and representative tumor areas free of necrosis and hemorrhage were premarked in the paraffin blocks. Consecutive sections (4μm) were then applied to 3-aminopropyltriethoxysilane-coated slides. After antigen retrieval in a microwave, the slides were incubated with a YMO1 monoclonal antibody (1:300) (Santa Cruz) for 30 min at room temperature or overnight at 4°C, followed by staining with a secondary antibody for 30 min, and then developed by 3, 3′- diaminobenzidine solution with counterstaining of hematoxylin. The slides were next evaluated by two pathologists in a blinded fashion. All IHC staining was independently assessed by two experienced pathologists. The staining intensity was graded from 0 to 2 (0, no staining; 1, weak; 2, strong). The staining extent was graded from 0 to 4 based on the percentage of immunoreactive tumor cells (0%, 1%-5%, 6%-25%, 26%-75%, 76%-100%). A score ranging from 0 to 8 was calculated by multiplying the staining extent score with the staining intensity score, resulting in a low (0-4) level or a high (6-8) level for each sample [34].

### Cell lines

L02 cells were obtained from the Cancer Research Institute of CSU. HepG2 cells were purchased from the American Type Culture Collection (ATCC, Rockville, MD). MHCC97-L, MHCC97-H and HCCLM3 cells were gifted from the Liver Cancer Institute of Fudan University (Shanghai, China). All cell lines were routinely maintained in the high glucose DMEM supplemented with 10% fetal bovine serum, 100 U/mL penicillin, and 100 mg/mL streptomycin at 37°C in a humidified incubator under 5% CO_2_.

### Plasmid constructs and transfection

The Full-length YMO1 coding region was obtained by RT-PCR amplification of normal human liver cDNA and then subcloned into a pcDNA3.1 vector. RhoC siRNA was purchased from GeneChem Corporation (Shanghai, China). YMO1 shRNA (5′-GGAGCTAACCCGGTATTTATT-3′) was construct by GeneChem Corporation (GeneChem, Shanghai, China). The cells (4×10^5^/well) were plated in 6-well plates and then transfected with either 4μg of pcDNA-YMO1 or pcDNA3.1 (control vector) using Lipofectamine LTX reagent (Invitrogen, Carlsbad, CA). Cells were next subjected to G418 selection 48h after transfection for 16 days.

### *In vivo* assays for tumorigenicity

HCCLM3 cells (1×10^7^cells) transfected with a pcDNA-YMO1 plasmid or a pcDNA3.1 vector were implanted subcutaneously into the left upper flank of a 4-week-old male Balb/c nude mouse (5/group). Tumor diameter was measured every 2-3 days for 4 weeks. Tumor volume (mm^3^) was estimated by measuring the longest and the shortest diameter of the tumor and calculated as follows: tumor volume (mm3) = (L × W^2^)/2, where L = long axis and W = short axis [35]. Then, the subcutaneous tumor tissues were removed and implanted into the livers of two groups of nude mice with eight mice in each group as follows. Subcutaneous tumors were harvested 35 days after implantation and cut into pieces of 1.0 mm^3^. One piece was then selected to implant into the left liver lobe of each mouse (8/group). The mice were sacrificed 4 weeks later to measure the size and weight of tumor. Mice with visible colonies around the local tumor were considered intrahepatic metastasis-positive. The lung tissue of each mouse was fixed, embedded, sectioned serially, stained with hematoxylin and eosin (H&E), and observed under a microscope. All experimental procedures were approved by the Animal Ethics Committee of the Central South University.

### Adhesion assay

For cell-cell adhesion assay: a 96-well plate was coated with fibronectin at 37°C for 1 h and washed twice with washing buffer (0.1% BSA in DMEM). Plates were blocked by blocking buffer (0.5% BSA in DMEM) at 37°C in a CO_2_ incubator for 60 min. HCC cells were then washed with washing buffer (0.1% BSA in DMEM). When the cell count reached 1×10^5^/mL, 100 ul cells were added into each well and cultured for 60, 90 or 120 min at 37°C. The medium was entirely removed and unbound cells were washed away with PBS. Cells were stained with 20uL MTT (5 mg/mL). Cell adhesion was quantified on a colorimetric ELISA plate reader Elx800 (Bio-Tek, Burlington, Vermont) at 570 nm. Differences in the Cell-ECM adhesion assay were: cells, instead of fibronectin, were used to adhere to 96-well culture plates to form the monolayer; and adherent cells were quantified using microscopy.

### Flow cytometry

To quantify cellular apoptosis, HCCLM3^Vector^, HCCLM3^YMO+^, HepG2^Vector^ and HepG2^shYMO1^ HCC cells were stained with an Annexin V-FITC/PI staining Kit (Bestbio, Beijing, China) according to the manufacturer's protocol and analyzed by flow cytometry. Three independent experiments were carried out.

### Confocal microscopy

Cells grown on coverslips were first fixed with 4% paraformaldehyde and then permeabilized in 0.5% TritonX-100. After blocking with 10% goat serum, the cells were stained with Phalloidin Rhodamine (Invitrogen) for F-actin. Nuclei were counterstained with DAPI. The cells were next examined under a confocal laser scanning microscope (Nikon, Tokyo, Japan) equipped with the appropriate filters for three-color imaging of cells with a CCD camera.

### Co-immunoprecipitation

The cells were solubilized in medium containing 1% NP-40, 0.5% deoxycholate (DOC) and SDS. Supernatants of cell lysates were then collected by centrifugation at 50,000 rpm for 10 min, followed by incubation with an YMO1 antibody overnight at 4°C. The immune complexes were next pulled-down by protein A-Sepharose beads. After washes, the protein complexes were eluted out by sample buffer containing 0.2 M DTT and then subjected to western blot analysis by probing with antibodies against Cdc42 (1:1000), Rac1 (1:1000), RhoA (1:1000), RhoC (1:1000), RhoGDI (1:1000) and mouse monoclonal antibody for Ubiquitin (1:1000, Santa Cruz Biotechnology, Santa Cruz, CA), respectively.

### GTPase pull-down assay

The GTPase activity for RhoC was measured using an Active Rho Pull-Down and Detection Kit (Pierce, Rockford, IL) as instructed. Lysates from serum-starved HCCLM3^vector^, HCCLM3^YMO1+^ and HCCLM3^YMO1+RhoC+^ cells were treated with GTPγS or GDP, respectively. Active Rho was then enriched by glutathione agarose resin according to the instruction. Each elution was next analyzed by western blotting using an Rho antibody. GTPγS- and GDP-labeled cell lysates were used as a positive and a negative control, respectively. Data are expressed as the percentage of Rho activity over HCCLM3^vector^.

### Dual luciferase reporter assay

The *YMO1* promoter (−1757 to +180, transcriptional starting site as +1) was subcloned into a pGL3 vector (pGL3-YMO1). A mutated YMO1 promoter was also constructed. In this construct, the putative PAX5 binding site at position −645 to −617 was disrupted (pGL3-YMO1-M). HCCLM3 cells were stably transfected with a pcDNA3-PAX5 or a pcDNA3.1 plasmid in 24-well plates along with a pGL3-YMO1 or a pGL3-YMO1-M plasmid by Lipofectamine LTX (Invitrogen). PGK Renilla was used as an internal control. The cells were harvested 48h after transfection for analysis of luciferase activities using a dual-luciferase reporter assay system (Promega, Madison, WI). Three independent assays were performed.

### Chromatin immunoprecipitation (ChIP) assay

The cells (3×10^6^) were harvested after 1 day of culture and then subjected to ChIP assay using a Pierce^®^ Chromatin Prep Module and Pierce^®^ Agarose ChIP Kit (Pierce, Rockford, IL). A mouse monoclonal Pax5 antibody (Santa Cruz) was used to pull-down the Pax5-DNA complex. The precipitates were then used as templates to amplify the *YMO1* promoter region containing the Pax5 binding site with following primers: forward, 5′-TTG GTG GTT GTA GAA GAT G-3′; reverse, 5′-ACA GGA GCA GAG GAA ATG-3′. Input DNAs from HCCLM3^vector^ and HCCLM3^PAX5+^ cells were used as positive controls. Precipitates resulted from a control IgG were used as negative controls.

### Mutations detecting of the Pax5-binding domain within the promoter of YMO1

PCR was performed at 95°C for 5 min followed by 30 cycles of 95°C for 30 sec, 60°C for 30 sec and 72°C for 50 sec with a final extension at 72°C for 10 min. The primer was as follows: forward, 5′-CAGCGGGAGAGACAAAAGTC-3′; reverse, 5′- GCTCAGCAACGGGTTATGTT -3′. After amplifying of the target band, sequences were detected (Biosune, Shanghai, China).

### Statistical analysis

All data were analyzed using the statistical software SPSS 17 for Windows (SPSS Inc., Chicago, IL). Comparisons were made for the differences in clinical and pathologic features. Spearman rank-correlation analysis was used to analyze the correlation between YMO1 expression and clinicopathological parameters. Survival curves were constructed using the Kaplan-Meier method and evaluated using the log-rank test. The Cox proportional hazard regression model was used to identify the risk factors that were independently associated with overall survival and disease-free survival. Continuous data were presented as mean ± SD and analyzed by Student-t test. Categorical data were analyzed with Fisher's exact test. All tests were two-sided and P<0.05 was considered statistically significant.

## SUPPLEMENTARY FIGURES AND TABLES



## References

[1] Torre LA, Bray F, Siegel RL, Ferlay J, Lortet-Tieulent J, Jemal A (2015). Global cancer statistics, 2012. CA: a cancer journal for clinicians.

[2] Hanahan D, Weinberg RA (2011). Hallmarks of cancer: the next generation. Cell.

[3] Xu J, Li X, Yang H, Chang R, Kong C, Yang L (2013). SIN1 promotes invasion and metastasis of hepatocellular carcinoma by facilitating epithelial-mesenchymal transition. Cancer.

[4] Wang W, Yang LY, Huang GW, Lu WQ, Yang ZL, Yang JQ, Liu HL (2004). Genomic analysis reveals RhoC as a potential marker in hepatocellular carcinoma with poor prognosis. British journal of cancer.

[5] Wu F, Yang LY, Li YF, Ou DP, Chen DP, Fan C (2009). Novel role for epidermal growth factor-like domain 7 in metastasis of human hepatocellular carcinoma. Hepatology.

[6] Sun CX, Robb VA, Gutmann DH (2002). Protein 4. 1 tumor suppressors: getting a FERM grip on growth regulation. Journal of cell science.

[7] Ruiz-Saenz A, van Haren J, Laura Sayas C, Rangel L, Demmers J, Millan J, Alonso MA, Galjart N, Correas I (2013). Protein 4. 1R binds to CLASP2 and regulates dynamics, organization and attachment of microtubules to the cell cortex. Journal of cell science.

[8] Wang J, Song J, An C, Dong W, Zhang J, Yin C, Hale J, Baines AJ, Mohandas N, An X (2014). A 130-kDa protein 4. 1B regulates cell adhesion, spreading, and migration of mouse embryo fibroblasts by influencing actin cytoskeleton organization. The Journal of biological chemistry.

[9] Xi C, Ren C, Hu A, Lin J, Yao Q, Wang Y, Gao Z, An X, Liu C (2013). Defective expression of Protein 4. 1N is correlated to tumor progression, aggressive behaviors and chemotherapy resistance in epithelial ovarian cancer. Gynecologic oncology.

[10] Perez-Janices N, Blanco-Luquin I, Tunon MT, Barba-Ramos E, Ibanez B, Zazpe-Cenoz I, Martinez-Aguillo M, Hernandez B, Martinez-Lopez E, Fernandez AF, Mercado MR, Cabada T, Escors D, Megias D, Guerrero-Setas D (2015). EPB41L3, TSP-1 and RASSF2 as new clinically relevant prognostic biomarkers in diffuse gliomas. Oncotarget.

[11] Yeh CN, Pang ST, Chen TW, Wu RC, Weng WH, Chen MF (2009). Expression of ezrin is associated with invasion and dedifferentiation of hepatitis B related hepatocellular carcinoma. BMC cancer.

[12] Yang J, Qin LX, Li Y, Ye SL, Liu YK, Gao DM, Chen J, Tang ZY (2005). Molecular cytogenetic characteristics of the human hepatocellular carcinoma cell line HCCLM3 with high metastatic potential: comparative genomic hybridization and multiplex fluorescence in situ hybridization. Cancer genetics and cytogenetics.

[13] Heasman SJ, Ridley AJ (2008). Mammalian Rho GTPases: new insights into their functions from *in vivo* studies. Nature reviews Molecular cell biology.

[14] Baines AJ, Lu HC, Bennett PM (2014). The Protein 4. 1 family: hub proteins in animals for organizing membrane proteins. Biochimica et biophysica acta.

[15] Song X, Yang J, Hirbawi J, Ye S, Perera HD, Goksoy E, Dwivedi P, Plow EF, Zhang R, Qin J (2012). A novel membrane-dependent on/off switch mechanism of talin FERM domain at sites of cell adhesion. Cell research.

[16] Laprise P, Lau KM, Harris KP, Silva-Gagliardi NF, Paul SM, Beronja S, Beitel GJ, McGlade CJ, Tepass U (2009). Yurt, Coracle, Neurexin IV and the Na(+), K(+)-ATPase form a novel group of epithelial polarity proteins. Nature.

[17] Hirano M, Hashimoto S, Yonemura S, Sabe H, Aizawa S (2008). EPB41L5 functions to post-transcriptionally regulate cadherin and integrin during epithelial-mesenchymal transition. The Journal of cell biology.

[18] Shin K, Fogg VC, Margolis B (2006). Tight junctions and cell polarity. Annual review of cell and developmental biology.

[19] Ishiuchi T, Takeichi M (2011). Willin and Par3 cooperatively regulate epithelial apical constriction through aPKC-mediated ROCK phosphorylation. Nature cell biology.

[20] Laprise P, Beronja S, Silva-Gagliardi NF, Pellikka M, Jensen AM, McGlade CJ, Tepass U (2006). The FERM protein Yurt is a negative regulatory component of the Crumbs complex that controls epithelial polarity and apical membrane size. Developmental cell.

[21] Wang W, Wu F, Fang F, Tao Y, Yang L (2008). Inhibition of invasion and metastasis of hepatocellular carcinoma cells via targeting RhoC *in vitro* and *in vivo*. Clinical cancer research: an official journal of the American Association for Cancer Research.

[22] Rossman KL, Der CJ, Sondek J (2005). GEF means go: turning on RHO GTPases with guanine nucleotide-exchange factors. Nature reviews Molecular cell biology.

[23] Wu M, Wu ZF, Rosenthal DT, Rhee EM, Merajver SD (2010). Characterization of the roles of RHOC and RHOA GTPases in invasion, motility, and matrix adhesion in inflammatory and aggressive breast cancers. Cancer.

[24] Li H, Peyrollier K, Kilic G, Brakebusch C (2014). Rho GTPases and cancer. BioFactors.

[25] Stankiewicz TR, Linseman DA (2014). Rho family GTPases: key players in neuronal development, neuronal survival, and neurodegeneration. Frontiers in cellular neuroscience.

[26] Fusella F, Ferretti R, Recupero D, Rocca S, Di Savino A, Tornillo G, Silengo L, Turco E, Cabodi S, Provero P, Pandolfi PP, Sapino A, Tarone G, Brancaccio M (2014). Morgana acts as a proto-oncogene through inhibition of a ROCK-PTEN pathway. The Journal of pathology.

[27] Fang F, Yang L, Tao Y, Qin W (2012). FBI-1 promotes cell proliferation and enhances resistance to chemotherapy of hepatocellular carcinoma *in vitro* and *in vivo*. Cancer.

[28] Yang Z, Yuan XG, Chen J, Luo SW, Luo ZJ, Lu NH (2013). Reduced expression of PTEN and increased PTEN phosphorylation at residue Ser380 in gastric cancer tissues: a novel mechanism of PTEN inactivation. Clinics and research in hepatology and gastroenterology.

[29] Xia L, Huang W, Tian D, Zhu H, Zhang Y, Hu H, Fan D, Nie Y, Wu K (2012). Upregulated FoxM1 expression induced by hepatitis B virus X protein promotes tumor metastasis and indicates poor prognosis in hepatitis B virus-related hepatocellular carcinoma. Journal of hepatology.

[30] Palmisano WA, Crume KP, Grimes MJ, Winters SA, Toyota M, Esteller M, Joste N, Baylin SB, Belinsky SA (2003). Aberrant promoter methylation of the transcription factor genes PAX5 alpha and beta in human cancers. Cancer research.

[31] Chang RM, Yang H, Fang F, Xu JF, Yang LY (2014). MicroRNA-331-3p promotes proliferation and metastasis of hepatocellular carcinoma by targeting PH domain and leucine-rich repeat protein phosphatase. Hepatology.

[32] Yang LY, Fang F, Ou DP, Wu W, Zeng ZJ, Wu F (2009). Solitary large hepatocellular carcinoma: a specific subtype of hepatocellular carcinoma with good outcome after hepatic resection. Annals of surgery.

[33] McShane LM, Altman DG, Sauerbrei W, Taube SE, Gion M, Clark GM, Statistics Subcommittee of the NCIEWGoCD (2005). REporting recommendations for tumor MARKer prognostic studies (REMARK). Nature clinical practice Urology.

[34] Liu Y, Zhang JB, Qin Y, Wang W, Wei L, Teng Y, Guo L, Zhang B, Lin Z, Liu J, Ren ZG, Ye QH, Xie Y (2013). PROX1 promotes hepatocellular carcinoma metastasis by way of up-regulating hypoxia-inducible factor 1alpha expression and protein stability. Hepatology.

[35] Zhang JF, He ML, Fu WM, Wang H, Chen LZ, Zhu X, Chen Y, Xie D, Lai P, Chen G, Lu G, Lin MC, Kung HF (2011). Primate-specific microRNA-637 inhibits tumorigenesis in hepatocellular carcinoma by disrupting signal transducer and activator of transcription 3 signaling. Hepatology.

