# Supplementary material for: YMO1 suppresses invasion and metastasis by inhibiting RhoC signaling and predicts favorable prognosis in hepatocellular carcinoma
Source: Oncotarget. 2016 Jul 27;7(34):55585–600. doi: 10.18632/oncotarget.10866 (PMC5342438; doi:10.18632/oncotarget.10866)
Supplement: Supplementary file 1 [file oncotarget-07-55585-s001.pdf]

# YMO1 suppresses invasion and metastasis by inhibiting RhoC signaling and predicts favorable prognosis in hepatocellular carcinoma

## SUPPLEMENTARY FIGURES AND TABLES

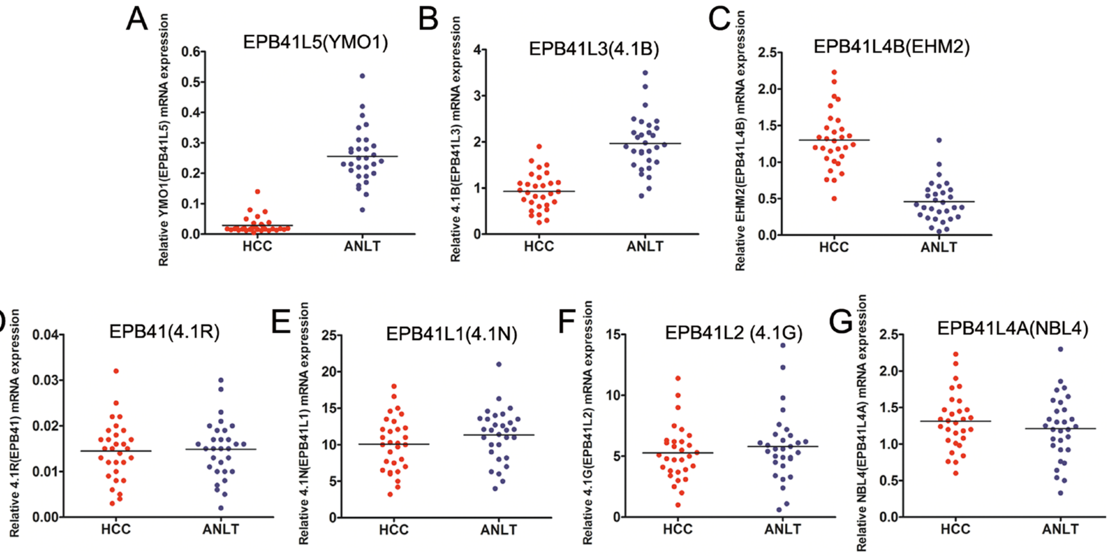


**Supplementary Figure S1: Expression of YMO1 and other 4.1 protein family in HCC and ANLT tissues.** The mRNA expression level of **A.** EPB41L5(YMO1), **B.** EPB41L3(4.1B), **C.** EPB41L4B(EHM2), **D.** EPB41(4.1R), **E.** EPB41L1(4.1N), **F.** EPB41L2 (4.1G) **G.** EPB41L4A(NBL4), in HCC tumor and ANLT were compared. Fold inductions were calculated using the formula $2^{-\Delta Ct}$. Median values are indicated by horizontal lines.

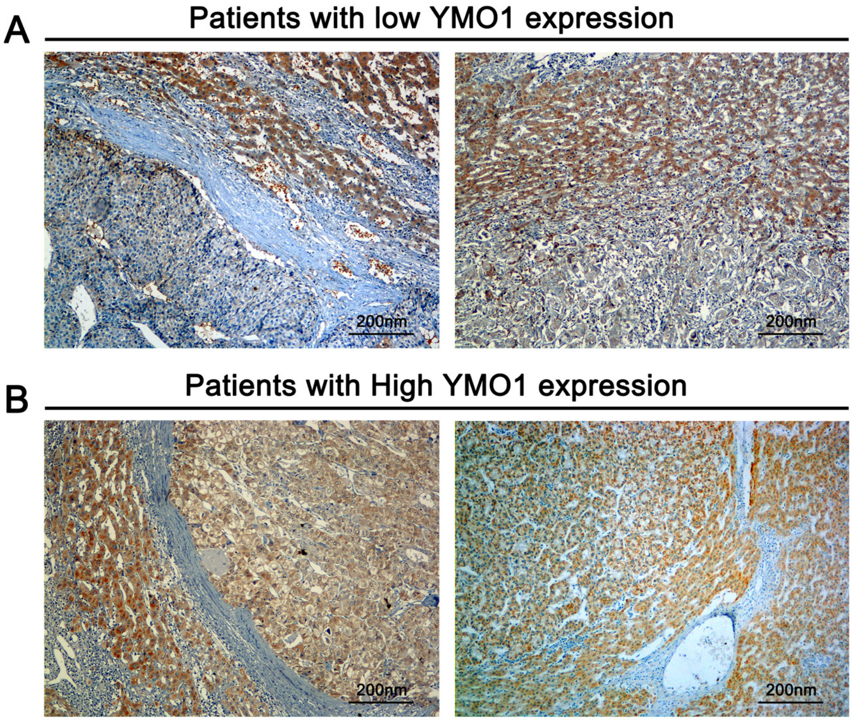


**Supplementary Figure S2: Representative images for YMO1 immunohistochemistry staining in the tumor borders.** YMO1 expression in HCC tissues is mostly lower than that in ANLT. Patients were divided into **A.** relative lower YMO1 expression group and **B.** relative higher YMO1 expression group.

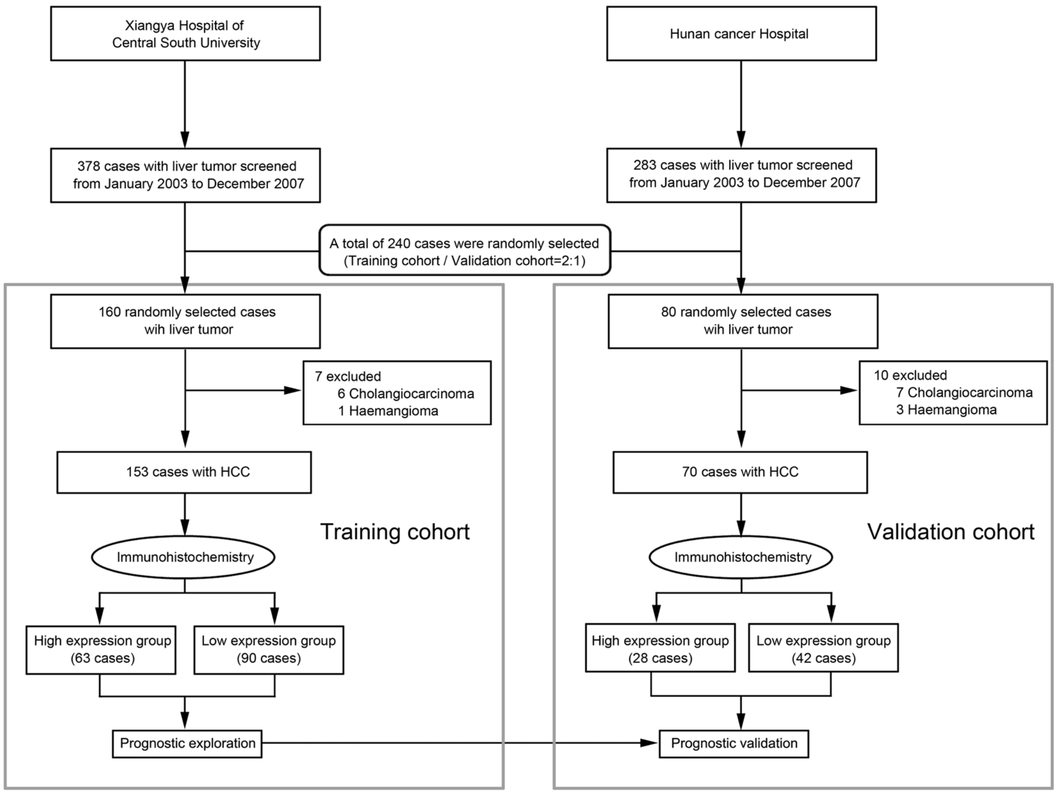


**Supplementary Figure S3: Flow diagram of patients included in study.**

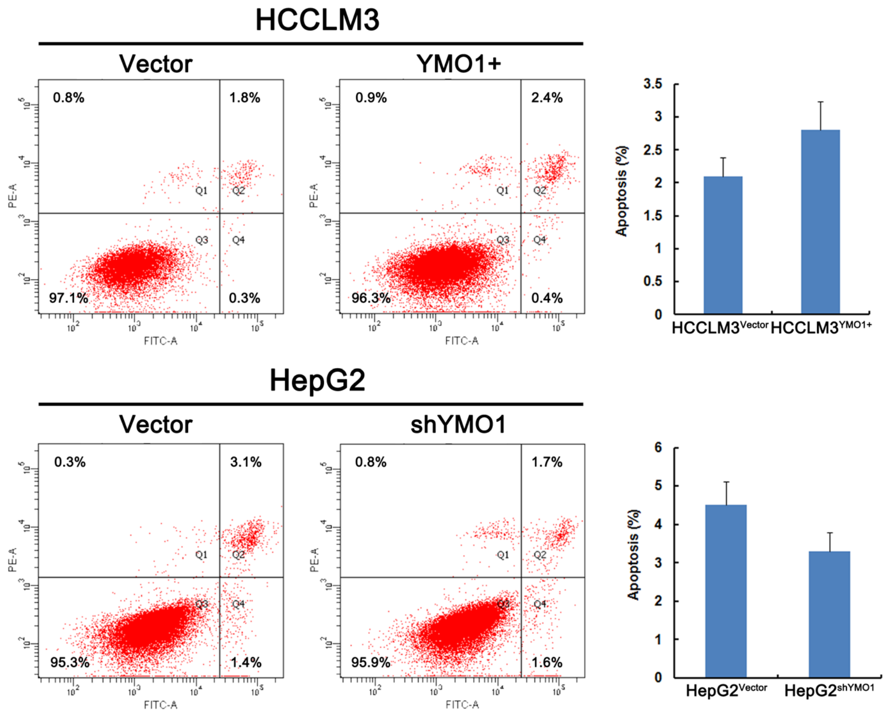


**Supplementary Figure S4: Overexpression or inhibition of YMO1 in HCCLM3 and HepG2 cell lines has no significant influence on apoptosis of HCC cells.**

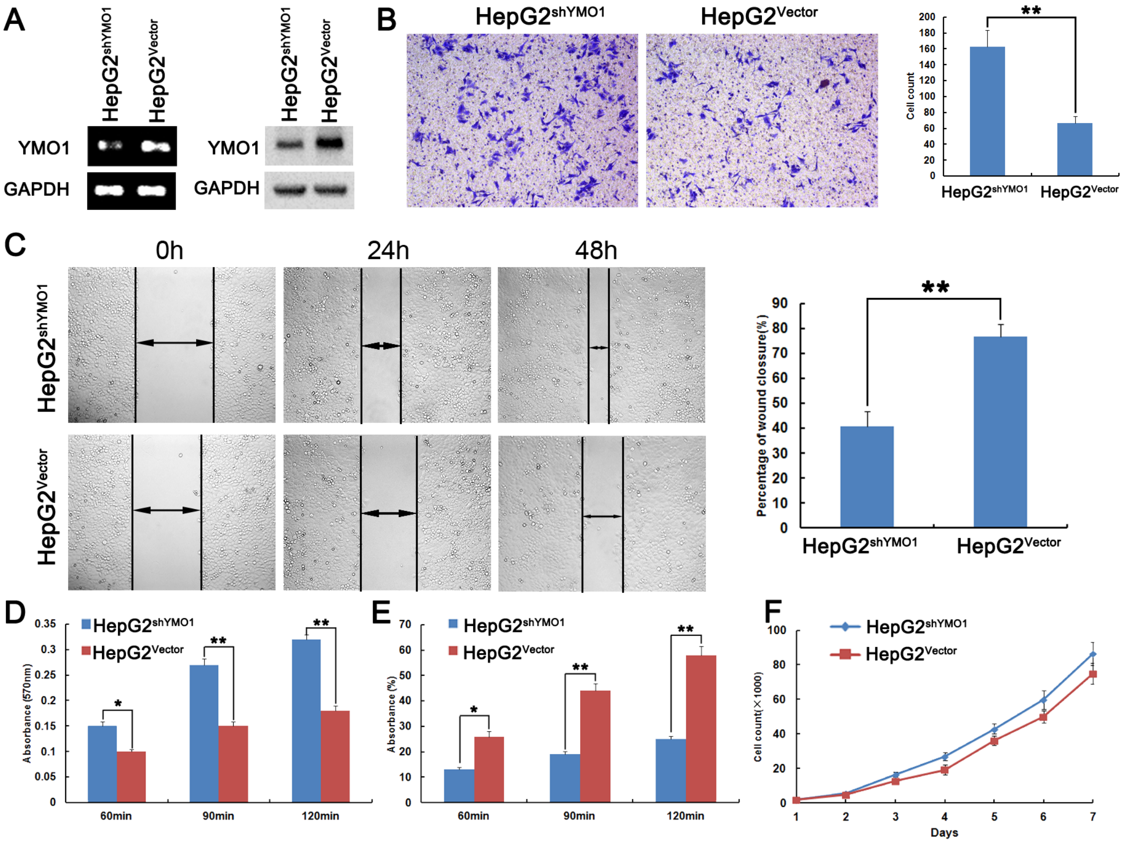


**Supplementary Figure S5: Inhibition of YMO1 promotes HCC cell migration and invasive potential *in vitro* and *in vivo*.** **A.** The expression efficiency of shYMO1 was evaluated by RT-PCR and Western blot analysis. **B.** The HepG2$^{shYMO1}$ and HepG2$^{Vector}$ cells that invaded through matrigel-coated transwell were stained with crystal violet. **C.** The HepG2$^{shYMO1}$ and HepG2$^{Vector}$ cells were scratched and wound closures were measured for 0, 24 and 48 hours culture. **D.** Cells were inoculated in fibronectin-coated plastic dishes and absorbance at 570 nm was measured. **E.** Monolayer $10^6$ cells were plated on monolayer of HepG2 cell. After incubation and elution, rate of adherence was measured. **F.** HCC cells were subjected to proliferation rate analysis. The cell numbers are the medians of 3 independent experiments (mean±SD). *, $P < 0.05$; **, $P < 0.01$.

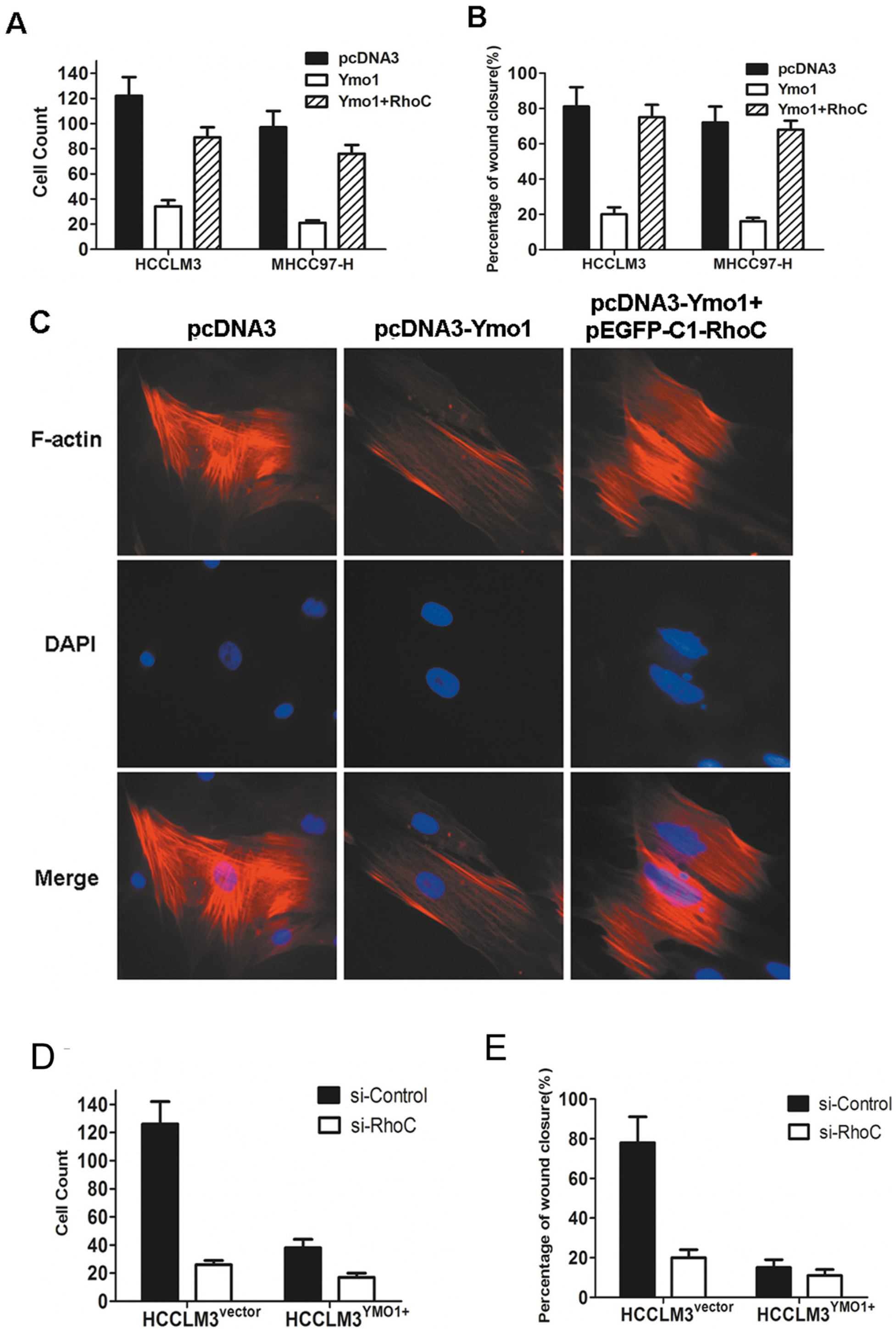


**Supplementary Figure S6: A.** Transwell assay for HCCLM3$^{Ymo1+}$ cells or MHCC97-H$^{Ymo1+}$ cells after co-transfected with pEGFP-C1-RhoC 3 days later. **B.** Wound healing assay for the migration of HCCLM3$^{Ymo1+}$ or MHCC97-H$^{Ymo1+}$ cells after exogenous overexpression of RhoC. **C.** Immunostaining of HCCLM3$^{vector}$, HCCLM3$^{Ymo1+}$ and HCCLM3$^{Ymo1+RhoC}$ cells. Images were acquired using confocal laser scaning microscopy. F-actin was stained with Phalloidin Rhodamine (red) and nucleus with DAPI (blue). Original magnification ×400. **D.** The transwell assay and **E.** wound healing assay result for HCCLM3$^{vector}$ and HCCLM3$^{Ymo1+}$ transfected with si-RhoC.

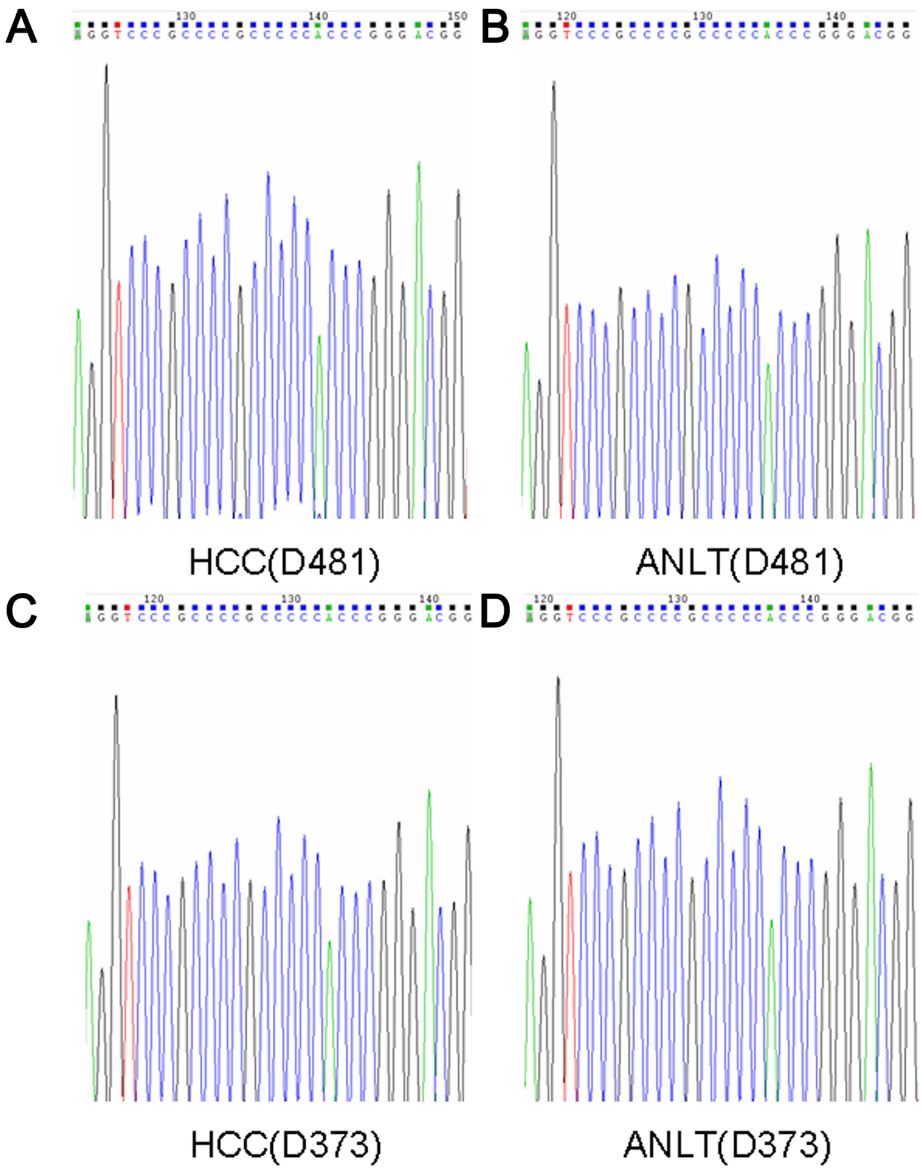


E

| | HCC | ANLT |
|---|---|---|
| Numbers of mutations in the Pax5-binding domain within the promoter of YMO1 | 0 | 0 |

**Supplementary Figure S7: No mutations of the Pax5-binding domain within the promoter of YMO1 was detected.** We detect 25 HCC specimens and the adjacent nontumoral liver tissues. **A-D.** The representative picture of sequencing results. **E.** Numbers of mutations in the Pax5-binding domain within the promoter of YMO1 in the 25 HCC specimens and the adjacent nontumoral liver tissues.

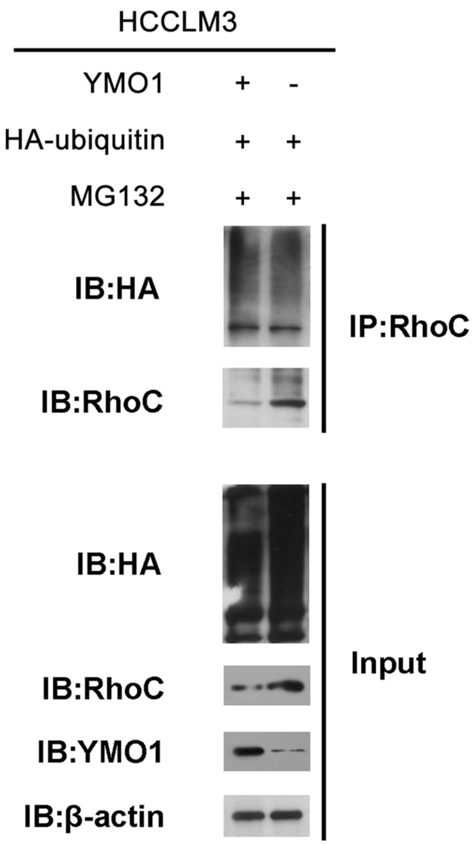


**Supplementary Figure S8: Ubiquitination of RhoC protein was detected in HCCLM3 cell line.** HCCLM3 cells were cotransfected with a pcDNA-YMO1 vector or control vector as well as a HA-ubiquitin vector. Before harvested the protein, the HCC cells were treated with 10μM MG132 for 6 h. Cell lysates were immunoprecipitated (IP) with RhoC antibody. The bound proteins and whole cell lysates were analyzed by immunoblotting (IB) with corresponding antibody.

**Supplementary Table S1: Clinicopathological characteristics of patients in training cohort and in validation cohort**

| Clinicopathologic variable | Counts | | *P* |
|---|---|---|---|
| | Training cohort | Validation cohort | |
| Gender | | | |
| Female | 26 | 12 | |
| Male | 127 | 58 | 0.978 |
| Age(year) | | | |
| ≤60 | 121 | 50 | |
| >60 | 32 | 20 | 0.210 |
| AFP | | | |
| <20 ng/ml | 50 | 23 | |
| ≥20 ng/ml | 103 | 47 | 0.979 |
| HBsAg | | | |
| Negative | 35 | 16 | |
| Positive | 118 | 54 | 0.998 |
| Liver cirrhosis | | | |
| Absence | 46 | 21 | |
| Presence | 107 | 49 | 0.992 |
| Tumor size(cm) | | | |
| ≤5 | 64 | 29 | |
| >5 | 89 | 41 | 0.955 |
| Tumor nodule number | | | |
| Solitary | 84 | 43 | |
| Multiple(≥2) | 69 | 27 | 0.361 |
| Capsular formation | | | |
| Presence | 89 | 29 | |
| Absence | 64 | 41 | 0.020 |
| Edmondson-Steiner grade | | | |
| I-II | 85 | 39 | |
| III-IV | 68 | 31 | 0.982 |
| Vascular invasion | | | |
| Absence | 95 | 31 | |
| Presence | 58 | 39 | 0.013 |
| TNM | | | |
| I | 74 | 31 | |
| II-III | 79 | 39 | 0.571 |
| BCLC staging | | | |
| 0-A | 81 | 27 | |
| B-C | 72 | 43 | 0.046 |
| Child-Pugh staging | | | |
| A | 103 | 37 | |
| B | 50 | 33 | 0.038 |

**Supplementary Table S2: The correlations of YMO1 with clinicopathological features of HCC in validation cohort**

| Clinicopathologic variable | YMO1 | | | |
|---|---|---|---|---|
| | n | Low expression | High expression | *P* |
| Gender | | | | |
| Female | 12 | 7 | 5 | |
| Male | 58 | 35 | 23 | 1.000 |
| Age(year) | | | | |
| ≤60 | 50 | 31 | 19 | |
| >60 | 20 | 11 | 9 | 0.589 |
| AFP | | | | |
| <20 ng/ml | 23 | 14 | 9 | |
| ≥20 ng/ml | 47 | 28 | 19 | 0.917 |
| HBsAg | | | | |
| Negative | 16 | 10 | 6 | |
| Positive | 54 | 32 | 22 | 0.816 |
| Liver cirrhosis | | | | |
| Absence | 21 | 12 | 9 | |
| Presence | 49 | 30 | 19 | 0.749 |
| Tumor size(cm) | | | | |
| ≤5 | 29 | 16 | 13 | |
| >5 | 41 | 26 | 15 | 0.488 |
| Tumor nodule number | | | | |
| Solitary | 43 | 20 | 23 | |
| Multiple(≥2) | 27 | 22 | 5 | **0.004** |
| Capsular formation | | | | |
| Presence | 29 | 13 | 16 | |
| Absence | 41 | 29 | 12 | **0.029** |
| Edmondson-Steiner grade | | | | |
| I-II | 39 | 21 | 18 | |
| III-IV | 31 | 21 | 10 | 0.238 |
| Vascular invasion | | | | |
| Absence | 31 | 12 | 19 | |
| Presence | 39 | 30 | 9 | **0.001** |
| TNM | | | | |
| I | 31 | 14 | 17 | |
| II-III | 39 | 28 | 11 | **0.024** |
| BCLC staging | | | | |
| 0-A | 27 | 17 | 14 | |
| B-C | 43 | 25 | 14 | 0.432 |
| Child-Pugh staging | | | | |
| A | 37 | 22 | 15 | |
| B | 33 | 20 | 13 | 0.922 |

**Supplementary Table S3: Univariate and multivariate analysis of factors associated with disease-free survival in training cohort**

| Variable | Univariate analysis | | | Multivariate analysis | |
|---|---|---|---|---|---|
| | n | RR(95%CI) | *P* | RR(95%CI) | *P* |
| Gender | | | | | |
| Female | 26 | 1 | | | |
| Male | 127 | 1.200(0769-1.873) | 0.422 | n.a. | n.a. |
| Age(year) | | | | | |
| ≤60 | 121 | 1 | | | |
| >60 | 32 | 1.079(0.705-1.651) | 0.726 | n.a. | n.a. |
| AFP | | | | | |
| <20 ng/ml | 50 | 1 | | | |
| ≥20 ng/ml | 103 | 0.802(0.573-1.122) | 0.783 | n.a. | n.a. |
| HBsAg | | | | | |
| Negative | 35 | 1 | | | |
| Positive | 118 | 1.047(0.722-1.517) | 0.810 | n.a. | n.a. |
| Liver cirrhosis | | | | | |
| Absence | 46 | 1 | | 1 | |
| Presence | 107 | 1.558(1.057-2.298) | **0.025** | 1.661(1.111-2.484) | **0.013** |
| Tumor size(cm) | | | | | |
| ≤5 | 64 | 1 | | | |
| >5 | 89 | 1.343(0.941-1.917) | 0.104 | n.a. | n.a. |
| Tumor nodule number | | | | | |
| Solitary | 84 | 1 | | 1 | |
| Multiple(≥2) | 69 | 1.515(1.068-2.150) | **0.020** | 1.683(1.179-2.404) | **0.004** |
| Capsular formation | | | | | |
| Presence | 89 | 1 | | 1 | |
| Absence | 64 | 1.572(1.108-2.29) | **0.011** | 1.432(1.002-2.047) | **0.049** |
| Edmondson-Steiner grade | | | | | |
| I-II | 85 | 1 | | | |
| III-IV | 68 | 1.297(0.916-1.835) | 0.143 | n.a. | n.a. |
| Vascular invasion | | | | | |
| Absence | 95 | 1 | | 1 | |
| Presence | 58 | 1.825(1.276-8.611) | **0.001** | 1.698(1.168-2.468) | **0.006** |
| TNM | | | | | |
| I | 74 | 1 | | | |
| II-III | 79 | 1.216(0.958-1.543) | 0.107 | n.a. | n.a. |
| BCLC staging | | | | | |
| 0-A | 81 | 1 | | | |
| B-C | 72 | 1.400(0.997-1.965) | 0.052 | n.a. | n.a. |
| Child-Pugh staging | | | | | |
| A | 103 | 1 | | | |
| B | 50 | 1.406(0.983-2.012) | 0.062 | n.a. | n.a. |
| YMO1 expression | | | | | |
| high | 63 | 1 | | 1 | |
| Low | 90 | 1.788(1.245-2.567) | **0.002** | 1.698(1.168-2.468) | **0.006** |

**Supplementary Table S4: Univariate and multivariate analysis of factors associated with overall survival in validation cohort**

| Variable | | Univariate analysis | | Multivariate analysis | |
|---|---|---|---|---|---|
| | n | RR(95%CI) | *P* | RR(95%CI) | *P* |
| Gender | | | | | |
| Female | 12 | 1 | | | |
| Male | 58 | 0.615(0.310-1.217) | 0.535 | n.a. | n.a. |
| Age(year) | | | | | |
| ≤60 | 50 | 1 | | | |
| >60 | 20 | 0.888(0.513-1.537) | 0.672 | n.a. | n.a. |
| AFP | | | | | |
| <20 ng/ml | 23 | 1 | | | |
| ≥20 ng/ml | 47 | 1.178(0.697-1.991) | 0.542 | n.a. | n.a. |
| HBsAg | | | | | |
| Negative | 16 | 1 | | | |
| Positive | 54 | 1.094(0.618-1.939) | 0.758 | n.a. | n.a. |
| Liver cirrhosis | | | | | |
| Absence | 21 | 1 | | | |
| Presence | 49 | 0.860 (0.500-1.478) | 0.584 | n.a. | n.a. |
| Tumor size(cm) | | | | | |
| ≤5 | 29 | 1 | | | |
| >5 | 41 | 1.209(0.727-2.010) | 0.464 | n.a. | n.a. |
| Tumor nodule number | | | | | |
| Solitary | 43 | 1 | | 1 | |
| Multiple(≥2) | 27 | 3.834( 2.170-6.774) | **<0.001** | 4.342( 2.210-8.529) | **<0.001** |
| Capsular formation | | | | | |
| Presence | 29 | 1 | | 1 | |
| Absence | 41 | 1.819(1.092-3.029) | **0.022** | 1.655(0.967-2.835) | 0.066 |
| Edmondson-Steiner grade | | | | | |
| I-II | 39 | 1 | | | |
| III-IV | 31 | 1.341(0.803-2.240) | 0.261 | n.a. | n.a. |
| Vascular invasion | | | | | |
| Absence | 31 | 1 | | 1 | |
| Presence | 39 | 1.806(1.077-3.026) | **0.025** | 2.262(1.152-4.442) | **0.018** |
| TNM | | | | | |
| I | 31 | 1 | | 1 | |
| II-III | 39 | 2.589(1.542-4.348) | **<0.001** | 2.300(1.281-4.129) | **0.005** |
| BCLC staging | | | | | |
| 0-A | 27 | 1 | | | |
| B-C | 43 | 0.887(0.625-1.722) | 0.735 | n.a. | n.a. |
| Child-Pugh staging | | | | | |
| A | 37 | 1 | | 1 | |
| B | 33 | 1.562(0.948-2.574) | 0.080 | 0.891(0.504-1.577) | 0.693 |
| YMO1 expression | | | | | |
| high | 28 | 1 | | 1 | |
| Low | 42 | 1.927(1.131-3.281) | **0.016** | 1.784(1.089-4.003) | **0.021** |

**Supplementary Table S5: Univariate and multivariate analysis of factors associated with disease-free survival in validation cohort**

| Variable | | Univariate analysis | | Multivariate analysis | |
|---|---|---|---|---|---|
| | n | RR(95%CI) | *P* | RR(95%CI) | *P* |
| Gender | | | | | |
| Female | 12 | 1 | | | |
| Male | 58 | 1.248(0.604-2.578) | 0.130 | n.a. | n.a. |
| Age(year) | | | | | |
| ≤60 | 50 | 1 | | | |
| >60 | 20 | 0.824(0.475-1.430) | 0.491 | n.a. | n.a. |
| AFP | | | | | |
| <20 ng/ml | 23 | 1 | | | |
| ≥20 ng/ml | 47 | 0.974(0.588-1.615) | 0.920 | n.a. | n.a. |
| HBsAg | | | | | |
| Negative | 16 | 1 | | | |
| Positive | 54 | 1.183(0.669-2.090) | 0.563 | n.a. | n.a. |
| Liver cirrhosis | | | | | |
| Absence | 21 | 1 | | | |
| Presence | 49 | 1.034(0.563-2.663) | 0.904 | n.a. | n.a. |
| Tumor size(cm) | | | | | |
| ≤5 | 29 | 1 | | | |
| >5 | 41 | 1.139(0.686-1.892) | 0.614 | n.a. | n.a. |
| Tumor nodule number | | | | | |
| Solitary | 43 | 1 | | 1 | |
| Multiple(≥2) | 27 | 3.627(2.055-6.400) | **<0.001** | 3.627(2.055-6.400) | **<0.001** |
| Capsular formation | | | | | |
| Presence | 29 | 1 | | | |
| Absence | 41 | 1.934(1.144-3.270) | **0.014** | 0.932(0.528-1.646) | 0.812 |
| Edmondson-Steiner grade | | | | | |
| I-II | 39 | 1 | | | |
| III-IV | 31 | 0.271(0.798-2.227) | 0.352 | n.a. | n.a. |
| Vascular invasion | | | | | |
| Absence | 31 | 1 | | 1 | |
| Presence | 39 | 1.754(1.596-6.901) | **0.001** | 1.689(1.308-4.487) | **0.016** |
| TNM | | | | | |
| I | 31 | 1 | | | |
| II-III | 39 | 2.587(1.550-4.318) | **<0.001** | 2.361(1.333-4.181) | **0.003** |
| BCLC staging | | | | | |
| 0-A | 27 | 1 | | | |
| B-C | 43 | 1.042(0.628-1.730) | 0.874 | n.a. | n.a. |
| Child-Pugh staging | | | | | |
| A | 37 | 1 | | 1 | |
| B | 33 | 1.596(0.968-2.633) | 0.067 | 1.061(0.932-1.646) | 0.808 |
| YMO1 expression | | | | | |
| high | 28 | 1 | | 1 | |
| Low | 42 | 1.858(1.091-3.165) | **0.023** | 1.812(1.041-3.157) | **0.036** |

**Supplementary Table S6: Correlation between PAX5 expression and clinicopathological characteristics**

| Variable | n | PAX5 expression | | *P* |
|---|---|---|---|---|
| | | Low expression (n=98) | High expression (n=55) | |
| Gender | | | | |
| Female | 26 | 15 | 11 | |
| Male | 127 | 83 | 44 | 0.458 |
| Age(year) | | | | |
| ≤60 | 121 | 77 | 44 | |
| >60 | 32 | 21 | 11 | 0. 835 |
| AFP | | | | |
| <20 ng/ml | 50 | 30 | 20 | |
| ≥20 ng/ml | 103 | 68 | 35 | 0.467 |
| HBsAg | | | | |
| Negative | 35 | 18 | 17 | |
| Positive | 118 | 80 | 38 | 0.076 |
| Liver cirrhosis | | | | |
| Absence | 46 | 29 | 17 | |
| Presence | 107 | 69 | 38 | 0.865 |
| Tumor size(cm) | | | | |
| ≤5 | 64 | 44 | 20 | |
| >5 | 89 | 54 | 35 | 0.304 |
| Tumor nodule number | | | | |
| Solitary | 84 | 46 | 38 | |
| Multiple(≥2) | 69 | 52 | 17 | **0.008** |
| Capsular formation | | | | |
| Presence | 89 | 47 | 42 | |
| Absence | 64 | 51 | 13 | **0.001** |
| Edmondson-Steiner grade | | | | |
| I-II | 85 | 49 | 36 | |
| III-IV | 68 | 49 | 19 | 0.065 |
| Vascular invasion | | | | |
| Absence | 95 | 52 | 43 | |
| Presence | 58 | 46 | 12 | **0.002** |
| TNM | | | | |
| I | 74 | 40 | 34 | |
| II-III | 79 | 58 | 21 | **0.013** |
| BCLC staging | | | | |
| 0-A | 81 | 51 | 30 | |
| B-C | 72 | 47 | 25 | 0.766 |

**Supplementary Table S7: Correlation analysis of YMO1, PAX5 and RhoC levels in HCC samples**

| | YMO1 expression | | *P* |
|---|---|---|---|
| | **Low (n = 90)** | **High (n = 63)** | |
| PAX5 expression | | | |
| Low (n=98) | 66 | 32 | 0.004 |
| High (n=55) | 24 | 31 | |
| RhoC expression | | | |
| Low (n=66) | 31 | 35 | 0.009 |
| High (n=87) | 59 | 28 | |
